# Separation of copper ions by nanocomposites using adsorption process

**DOI:** 10.1038/s41598-020-80914-w

**Published:** 2021-01-18

**Authors:** Nasim Danesh, Mohsen Ghorbani, Azam Marjani

**Affiliations:** 1 Department of Chemistry, Arak Branch, Islamic Azad University, Arak, Iran; 2 Faculty of Chemical Engineering, Babol Noshirvani University of Technology, Shariati St., Babol, 4714871167 Iran; 3 Department for Management of Science and Technology Development, Ton Duc Thang University, Ho Chi Minh City, Viet Nam; 4 Faculty of Applied Sciences, Ton Duc Thang University, Ho Chi Minh City, Viet Nam

**Keywords:** Chemistry, Engineering, Mathematics and computing

## Abstract

In this research, a novel nanocomposite adsorbent, graphene oxide modified with magnetite nanoparticles and Lauric acid containing ethylenediaminetetraacetic acid (GFLE) has been applied for the eliminate of Cu^2+^ ions. Adsorption performance was considered as a function of solution pH, Cu^2+^ ions concentration (C _Cu_^2+^), and temperature (T) and contact time (t). The levels of each variable were statistically optimized by Central Composite Design (CCD) and the response surface methodology (RSM) procedure to enhance the yield of system design. In these calculations, Y was measured as the response (the secondary concentration of Cu^2+^ ions in mg L^−1^). Highest copper adsorption occurred at time of 105 min, temperature of 40 °C, the initial concentration of 280 mg L^−1^, and pH = 1. The sorption equilibrium was well demonstrated using the Freundlich isotherm model. The second-order kinetics model suggested that the sorption mechanism might be ion exchange reactions. Thermodynamic factors and activation energy values displayed that the uptake process of Cu^2+^ ions was spontaneous, feasible, endothermic and physical in nature. Regeneration studies also revealed that GFLE could be consistently reused up to 3 cycles.

## Introduction

The impurity of water by toxicant heavy metals have significant impacts on different environmental life cycles and public health due to non-biodegradability, metal ion accumulation, and their quantities^1–4^. The most common hazardous heavy metals affecting human and environment are Sb, Cr, Cd, Cu, Pb, and Hg, etc.^1,3,5,6^. Copper can be found mainly as divalent cation in aqueous solutions and the most widely used metal ion in different industries, include metal finishing, paint, pigment industries, fertilizer, wood manufacturing and electrical^1,7^. Copper is necessary to people life and physical condition of the body, however, Cu^2+^ ion concentration levels more the tolerance limit will reason serious impacts on living organisms and the environment. The permissible limit for Cu^2+^ ion in industrial sewages, as proposed through the US EPA is 1.3 mg L^−1^, and long-term exposure makes provocation of eyes, nose, and mouth, stomachache, lung cancer, and neurotoxicity^3,8^.

The numerous method have been employed to remove Cu^2+^ from industrial wastes, such as liquid–liquid extraction, biosorption, chemical precipitation, ion exchange, electrodialysis, etc^1,3,9^. Which are usually expensive and have inherent limitations^2,9^. The adsorption process by the chelating characteristics of adsorbents is arguably one of the best techniques for elimination of heavy metals which has attracted significant notice because of simplicity, inexpensive, effectiveness and flexibility in design and action^3,9^. Various adsorbents have been employed for Cu^2+^ elimination including Nano-alumina, nanomagnets coated by EDTA, carbon nanotubes and hydroxyapatite nanoparticles providing high uptake efficiency^9^. Ethylenediaminetetraacetic acid (EDTA) is a hexadentate ligand and a well-known chelating agent with both carboxylate and amine functions providing strong metal-complexing behavior^10^. Chelating magnetic nanoparticles are classified as a notable classification of adsorbents due to their incomparable advantage of easy separation from solution via an external magnetic field which decreases the cost of industrial utilization and prohibits the treated water to be re-contamination^7,10^.

Optimization of Cu^2+^ adsorption process with classical techniques includes changing one independent parameter (pH, Cu^2+^ concentration, temperature and time) while retaining all others at a fixed level which is plenty of time to consume and costly. To solve this problem, response surface methodology (RSM) can be utilized to improve the adsorption of Cu^2+^ ions according to which, the effects of two or more factors can be studied simultaneously reducing the number of experiments.

In the present research, we investigate the combined impact of pH, Cu^2+^ concentration, temperature and time on Cu^2+^ ions adsorption from aqueous solution using magnetite graphene oxide/Lauric acid which contain Ethylenediaminetetraacetic acid nanoparticles (GFLE) which have been examined by central composite design (CCD) in RSM via Design Expert. The thermodynamic, kinetics and isotherm parameters for the adsorption Cu^2+^have also been computed and discussed.

## Materials and methods

### Materials

The chemical reagents in the present investigate involved^11^ and copper (II) nitrate (Cu (NO_3_)_2_, 99.5%), Merck. For the experiments, the source solution with concentration of 1 g L^−1^ copper was provided by disbanding determined values of copper nitrate in DI water to prepare the solutions. The solutions with concentrations between 60 to 500 mg L^−1^ was made by diluting the source solution. pH values, balancing of 1.0 to 5.0 by 0.1 M HCl and 0.1 M NaOH.

Studying the functional group in the nanocomposite was done with the help of FTIR; using a Bruker-Tensor 27 IR equipment in 400–4000 cm^−1^ with 2 cm^−1^ resolution. Surface morphology and size distribution of the nanoadsorbents was performed using TEM (Zeiss, EM10C, 80KV). Elemental analysis of the nanocomposite before and after absorption were done using EDX a Sirius SD microscope. X-ray diffraction were carried out using a Philiphs X^`^ Pert MPD X-ray system with Co k_α_ (λ = 1.78901 Å) (Holland) at room temperature.Buck Scientific atomic absorption spectrometer (Model-Buck 200 Series AA) was used to determine the concentrations of Cu^2+^ at 324.7 nm.

### Preparation of GFLE nanocomposite

**Figure 1:**
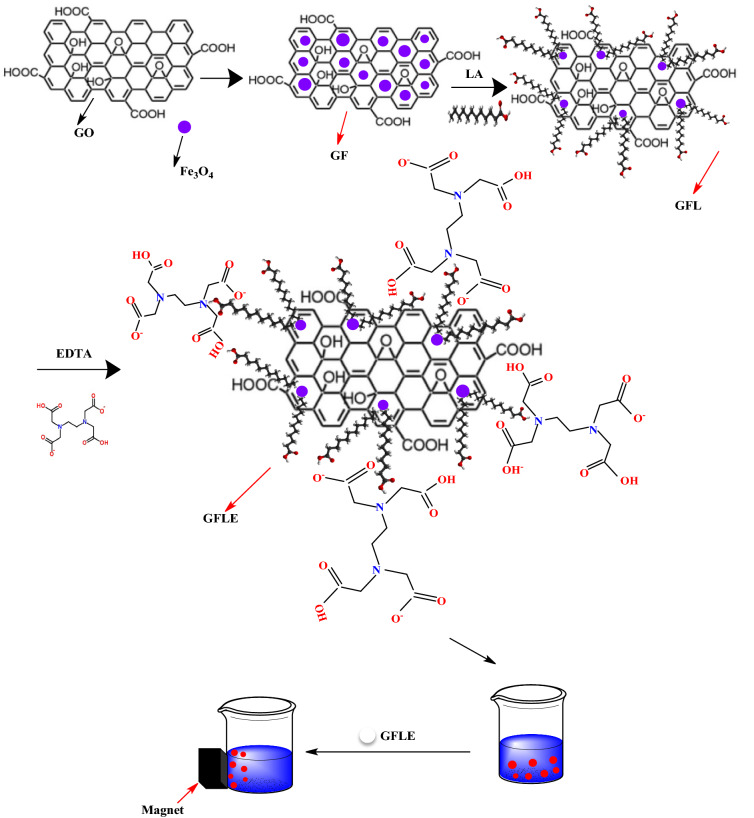
Multistep process of GFLE nanocomposite preparation and Cu(II) ions absorption process.

GO was made from graphite powder using the modified Hummers technique^12^. The GFLE nanocomposite was obtained via a sequential co-precipitation method shown in Fig. 1^11^.

### Batch adsorption experiments

For investigate the uptake efficiency of Cu^2+^ onto GFLE nanoadsorbent batch method was applied. 0.01 g of GFLE adsorbent was mixed with 10 mL samples solutions of different initial concentration (C_0_) from 60 to 500 (mg L^−1^), and shaken for contact times of 30 to 180 min at 300 rpm and different temperatures of 20 to 60 °C. Finally, the adsorbent was separated from the solution using a permanent magnet and the equilibrium concentration of Cu^2+^ was determined by AAS. The amount of Cu^2+^ adsorbed onto GFLE and the uptake percentage was exhibited as:

1 $$q_{t} = \frac{{(C_{0} - C_{e} )V}}{m}$$

2 $${\text{Uptake percentage}}\left( \% \right) = \frac{{C_{0} - C_{e} }}{{C_{0} }} \times 100$$

In which, q_t_ (mg g^−1^) is the adsorbed quantity of adsorbate per unit mass of the adsorbent at time t. concentrations C_0_ and C_e_ (mg L^−1^) are the initial and equilibrium of contaminants, respectively. m(g) is mass of the adsorbent and V (L) is the volume of adsorption solution^13^.

### Central composite method and design of analysis

The association between independent variables and response function (residual concentration or secondary concentration (was created by experimental mathematical models based on the RSM^7^. The optimum situation for the adsorption of Cu^2+^ by GFLE was defined using CCD under RSM^14^.

CCD analysis is used for high range prediction within the design range as well as outside the design range. A five-level four-selective parameter (pH, C_0 Cu_^2+^, t and T) are represented by X_1_, X_2_, X_3,_ and X_4_, respectively and the total of 30 testes were done (Table 1) inclusive six center points for repetition^29^. Residual concentration (Secondary concentration of Cu^2+^, Y) was known as the response. Empirical data achieved from the CCD model experiences can be studied in the form of the following equation ^11^:

3 $${\text{Y}} = {\upbeta }_{{0}} + \sum\limits_{{{\text{i}} = {1}}}^{{\text{k}}} {{\upbeta }_{{\text{i}}} {\text{x}}_{{\text{i}}} + \sum\limits_{{{\text{i}} = {1}}}^{{\text{k}}} {{\upbeta }_{{{\text{ii}}}} {\text{x}}_{{\text{i}}}^{{2}} + \sum\limits_{{{\text{i}} = {1}}}^{{{\text{k}} - {1}}} {\sum\limits_{{{\text{j}} = {2}}}^{{\text{k}}} {{\upbeta }_{{{\text{ij}}}} {\text{x}}_{{\text{i}}} {\text{x}}_{{\text{j}}} + {\upvarepsilon }} } } }$$

**Table 1:** Empirical range and levels of independent parameters.

Independent versus code		Range and level				
		− 2	− 1	0	+ 1	+ 2
pH	X_1_	1	2	3	4	5
The initial concentration of copper (mg L^−1^)	X_2_	60	170	280	390	500
Time (min)	X_3_	30	67.50	105	142.50	180
Temperature (°C)	X_4_	20	30	40	50	60

The Y demonstrates the magnitude of the response, β_0_, β_ii_, β_i_ and β_ij_ are the intercept term, the linear, the squared and the interplay affect, respectively. X_i_ and X_j_ are levels of the independent parameters and Ɛ displays the error^13^.

### Modeling of adsorption kinetics, isotherms, and thermodynamics

Three kinetics models have been selected to characterize the absorption performance of Cu^2+^ on nanoadsorbent, including Lagergren pseudo-first order, pseudo-second order^13^ and Second-order^15^ equations. All kinetic equations are provided in Table 2, where C_t_ and C_0_ are the concentration (mg dm^−3^) of Cu^2+^ at time and initial of the experiment, respectively. k_2_ is the second-order adsorption rate constant (L mg^−1^ min^−1^), kʹ_2_ is the pseudo-second order rate constant (g mg^−1^ min^−1^), k_1_ is the Lagergren pseudo-first order rate constant (min^−1^) and q_e_ and q_t_ are the uptake capacity (mg g^−1^) at equilibrium and at t (min), respectively^13,15^.

**Table 2:** Numerical equations in Cu (II) uptake kinetics.

Kinetic models	Linear equations		Graph	Calculated coefficients
Lagergren pseudo-first-order	$\ln (q_{e} - q_{t} ) = \ln q_{e} - k_{1} t$	(4)	ln(q_e_ − q_t_) *vs*. t	k_1_ = − slope, q_e_ = eintercept
Pseudo-second-order	$\frac{t}{{q_{t} }} = \frac{1}{{k^{\prime}_{2} q_{{e^{{}} }}^{2} }}_{{}} + \frac{t}{{q_{e} }}$	(5)	t/q_t_ *vs*. t	kʹ_2_ = slope^2^/intercept, q_e_ = 1/slope
Second-order	$\frac{1}{{C_{t} }} = k_{2} t + \frac{1}{{C_{0}^{{}} }}$	(6)	1/C_t_ versus t	K_2_ = slope

Adsorption isotherms are powerful tools which provide beneficial data about the mechanism, characteristics and the responsiveness of adsorbent into Cu^2+^ ions. In this study, Freundlich^7^, Langmuir, Temkin^7^ and Redlich–Peterson^16^. The Freundlich isotherm model is represented via the Eq. (7):

7 $$q_{e} = K_{f} C_{e}^{n}$$

q_e_ is the value of ions adsorbed per unit mass of the adsorbent (mg g^−1^) and C_e_ is the equilibrium concentration of Cu^2+^ ions. K_F_ and n are Freundlich constants, where K_F_ (mg g^−1^ (L mg^−1^) ^1/n^) is the sorption capacity of the adsorbent and n giving an emblem of how favorable the adsorption process is. In Freundlich isotherm, amounts of n bigger than 1 correspond to a favorable uptake system^7^.

The Langmuir adsorption isotherm describes adsorption processes forming monolayers onto nanocomposite with coverage homogeneous surface within the adsorbent^17^. The Langmuir equation can be represented as:

8 $$\frac{{C_{e} }}{{q_{e} }} = \frac{1}{{(bq_{m} )}} + \frac{{C_{e} }}{{q_{m} }}$$

The C_e_ is the equilibrium concentration (mg L^−1^), q_m_ is the maximum sorption capacity of the adsorbent for the elimination of Cu^2+^ ions (mg g^−1^) and b is the isotherm parameter in L mg^−116^. The Temkin model of isotherm is assigned to illustrate uptake potential among adsorbate/adsorbate; the heat of sorption for all the molecules in the layer would reduction linearly with covering. The linearized form of Temkin isotherm is displayed as:

9 $$q_{e} = B\ln A + B\ln C_{e}$$

10 $$B = \frac{RT}{{b_{t} }}$$

In which, A is the equilibrium binding constant (m g^−1^) and b_t_ is associated with the heat of uptake (kJ mol^−1^). The magnitudes of b_t_ and A were achieved from the slope and intercept of the plot q_e_ versus lnC_e_^13^.

The Redlich–Peterson isotherm is based on the supposition that the mechanism of sorption is a hybrid Langmuir and Freundlich isotherms. It contains “three parameter equation,” which it can be obtained using the following equations:

11 $$q_{e} = \frac{{K_{R} C_{e} }}{{1 + a_{R} C_{e}^{B} }}$$

$K_{R}$ (L g^−1^) and $a_{R}$ (mg^−1^) are the Redlich–Peterson isotherm constants. Also constant $\beta$ is a representative that lies between 0 and 1^16^.

Values of thermodynamic factors inclusive Gibbs free energy change (ΔG^o^), enthalpy change (ΔH^o^) and entropy change (ΔS^o^) perform the main role in the feasibility and orientation of the physicochemical sorption process of Cu^2+^ ions adsorption onto GFLE. The thermodynamic parameters can be written as equation ^16^:

12 $$\Delta G^\circ = - RT\ln K_{d}$$

13 $$\Delta G^{o} = \Delta H^{o} - T\Delta S^{o}$$

14 $$\ln K_{d} = \frac{{\Delta S^{o} }}{R} - \frac{\Delta H^\circ }{{RT}}$$

15 $$K_{d} = \frac{{q_{e} }}{{C_{e} }}$$

K_d_ is the distribution coefficient which depends on metal ion concentration and temperature, T is the T (K) and R is gas constant (8.314 J mol^−1^ K^−1^). ΔH^o^ and ΔS^o^ values are determined from the slope and intercept of ln K_d_ verses 1/T plot^13^.

### Activation energy

For investigate the physical or chemical nature of sorption, the activation energy of Cu^2+^ions onto GFLE adsorbent was expressed through a modified Arrhenius equation that describes sticking probability (S*) to surface coating (θ) was estimated as follow^17,19^:

16 $$s^{ * } = (1 - \theta )e^{{ - ({\raise0.7ex\hbox{${E_{a} }$} \!\mathord{\left/ {\vphantom {{E_{a} } {RT}}}\right.\kern-\nulldelimiterspace} \!\lower0.7ex\hbox{${RT}$}})}}$$

17 $$\theta = \left[ {1 - \frac{{C_{e} }}{{C_{0} }}} \right]$$

Values of E_a_ and S^*^ can be presented from the slope and intercept of ln (1 − θ) versus 1/T at three specified T of 293, 313, and 333 K.

### Desorption analysis

Desorption analysis was accomplished to calculate the regeneration capacity of the adsorbent. After adsorption step, Cu^2+^ ions on GFLE (0.01 g mL^−1^) were filtered, dried, weighed and shaken with 10 mL of desorbing agents (0.2 M, Na_2_EDTA) in 50 mL Erlenmeyer flasks at 300 rpm. After the solution had reached equilibrium, the C _Cu_^2+^ desorbed was calculated by the AAS. The above experiment was sequential three times under the same adsorption conditions.

### Error analysis

In order to check the isotherm and kinetic models, the chi-square test was applied in this paper to ascertain the best-fitted model for explaining the empirical data. The chi-square test can be represented as^17^:

18 $$\chi^{2} = \sum\limits_{i = 1}^{p} {\frac{{(q_{\exp } - q_{calc} )^{2} }}{{q_{\exp } }}}$$

where q_exp_ and q_calc_ (mg g^−1^) are determining ion concentration and ion concentration with isotherm and kinetic models. p indicant the number of experimental data, respectively. If information from the model were like to the empirical information, χ^2^ will be a minimum magnitude; then, χ^2^ will be a maximum magnitude.

## Results and discussion

### TEM and SEM analysis

**Figure 2:**
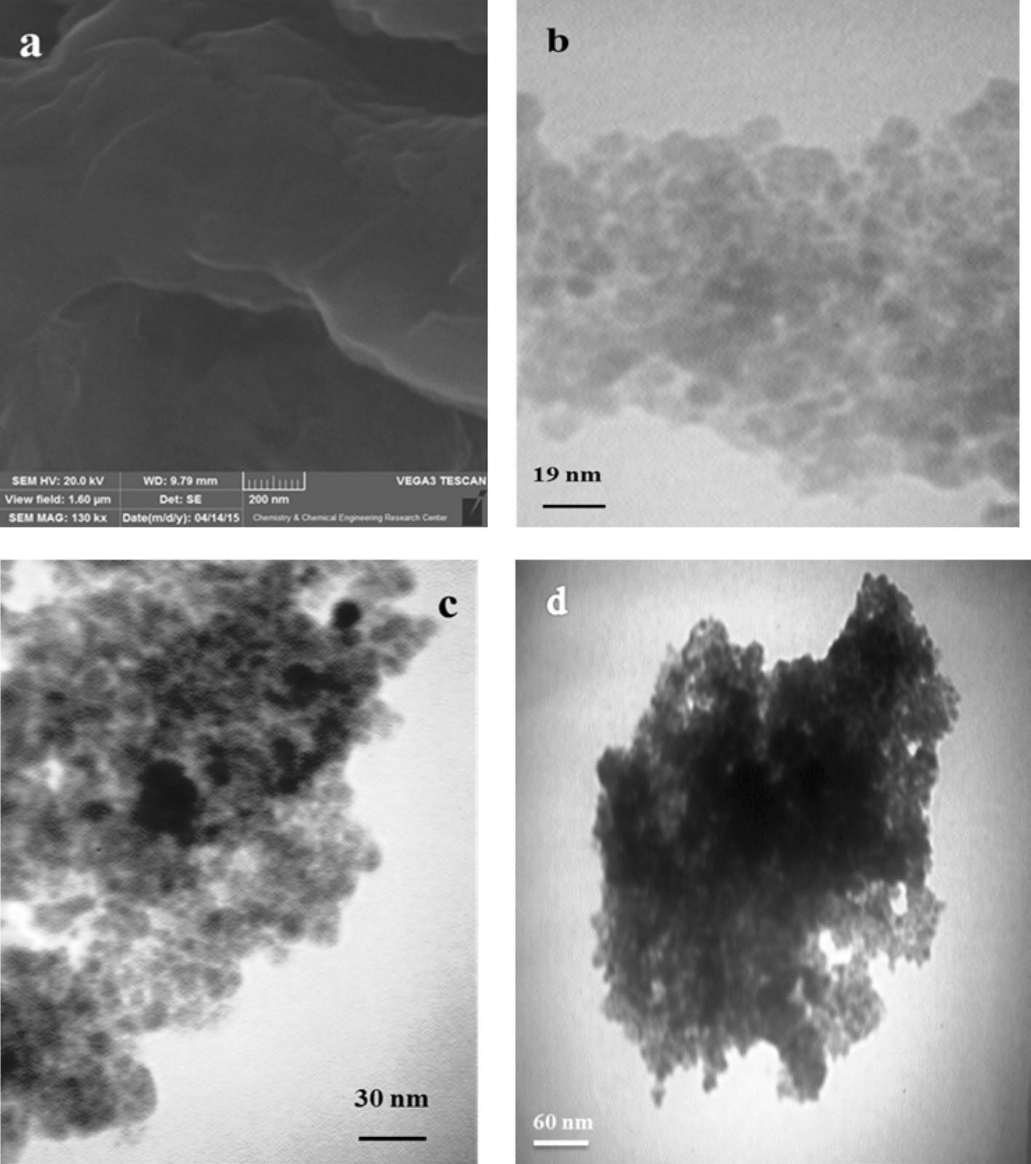
(**a**) SEM GO and TEM figures of (**b**) GF, (**c**) GFL and (**d**) GFLE.

Figure 2a–d displays morphology and structure of samples. SEM image of graphene oxide with rippled structure and full of wrinkled on the surface GO show in Fig. 2a. The TEM image of GF demonstrates in Fig. 2b, those spherical Fe_3_O_4_ particles homogeneously distribution on the surface of GO sheets. As seen from image in Fig. 2c, the dark background related good interactions between GO, Fe_3_O_4_ and Lauric acid, confirmed that the GFL nanocomposites was synthesized. As shown in Fig. 2d, GFLE nanoparticle had assembled turning into bigger size gathers which this phenomenon indicated a strong interaction occurred between Fe_3_O_4_ nanoparticles and the adjoining particles and the correction of Lauric acid and ethylenediaminetetraaceticacid, respectively.

### FTIR and XRD analysis

**Figure 3:**
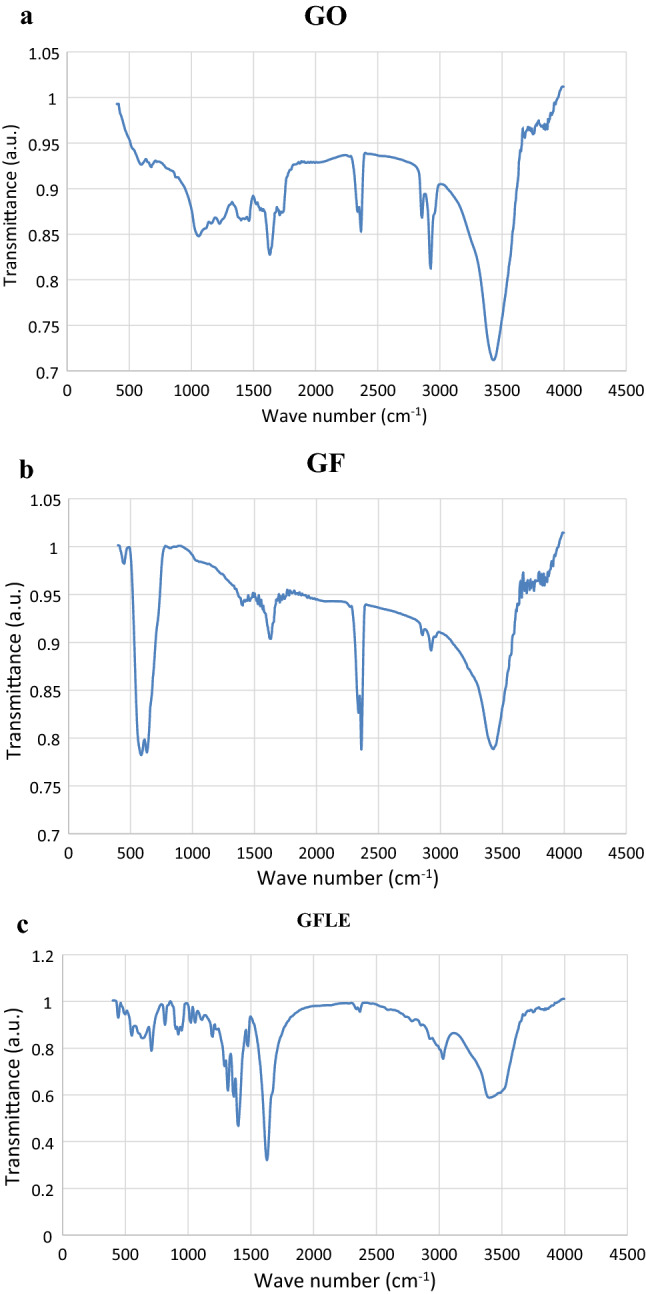
FTIR spectrum of (**a**) GO, (**b**) GF and (**C**) GFLE.

In the FT-IR analysis of GO, GF and GFLE are shown in Fig. 3. In Fig. 3c display the characteristic bands absorption of the bands of alkoxy C–O (1049 cm^−1^), C=O (1727 cm^−1^), epoxy C–O (1220 cm^−1^) and aromatic C=C (1622 cm^−1^). The peaks at 1253 and 3432 cm^−1^ was attributed to the stretching and bending vibrations of O–H, respectively. The structure of GFLE was endorsed by FT-IR graph as shown in Fig. 3c. Figure 3a is related the GO and show two absorption at 1731 and 3420 cm^−1^ corresponding to the attendance of C=O and O–H, respectively. Figure 3a The FT-IR spectrum of Fe_3_O_4_ with two peaks at 582 and 626 cm^−1^ were appointed to Fe–O stretching vibrations as exhibited in Fig. 3b. The characteristic peaks of the carboxylate anion at 1401 and 1627 cm^−1^ demonstrates which the ethylenediaminetetraacetic acid ligand was fixed onto iron oxide surface through the carboxylate anion. Peaks located at 2863 cm^−1^ and 2937 cm^−1^ are associated to methylene symmetric and asymmetric, respectively, in the structure of lauric acid. Band at 1048 cm^−1^ is related to C–N stretching of ethylenediaminetetraacetic acid are displayed in Fig. 3c^11^.

**Figure 4:**
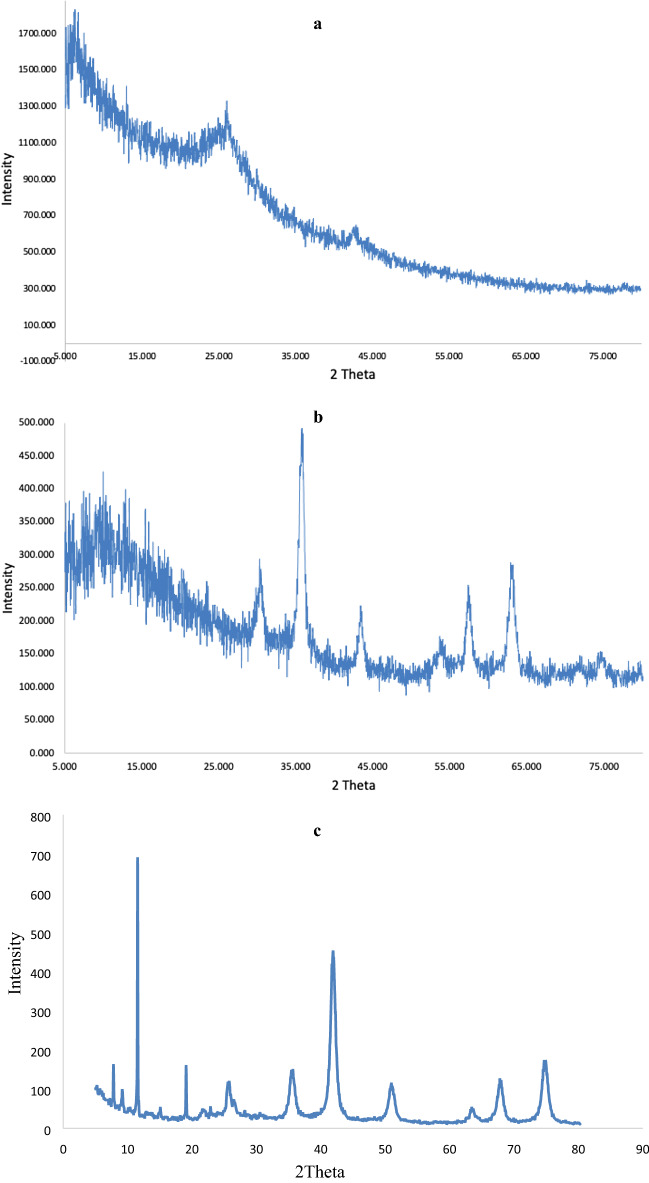
XRD pattern of (**a**) GO, (**b**) GF and (**C**) GFLE nanocomposite.

Figure 4 demonstrates the XRD analysis of GO, GF and GFLE nanocamposite. The peak at 2θ = 11.43°, assigned to the (001) surface of GO and the characteristic diffraction peaks at 2θ = 19.01°, 35.51°, 42.08°, 50.90°, 63.46°, 67.77°, 74.89°, 76.59° and 78.63° which correspond to (111), (220), (311), (400), (422), (511), (440), (620) and (622) crystal planes of Fe_3_O_4_ (JCPDS Card No. (79 - 0417)). Also, the peak indexed as plane (020) at 2θ = 25.58° could be corresponded to the/crystalline structure of EDTA cross-linked Lauric acid^11^.

### BET results

**Figure 5:**
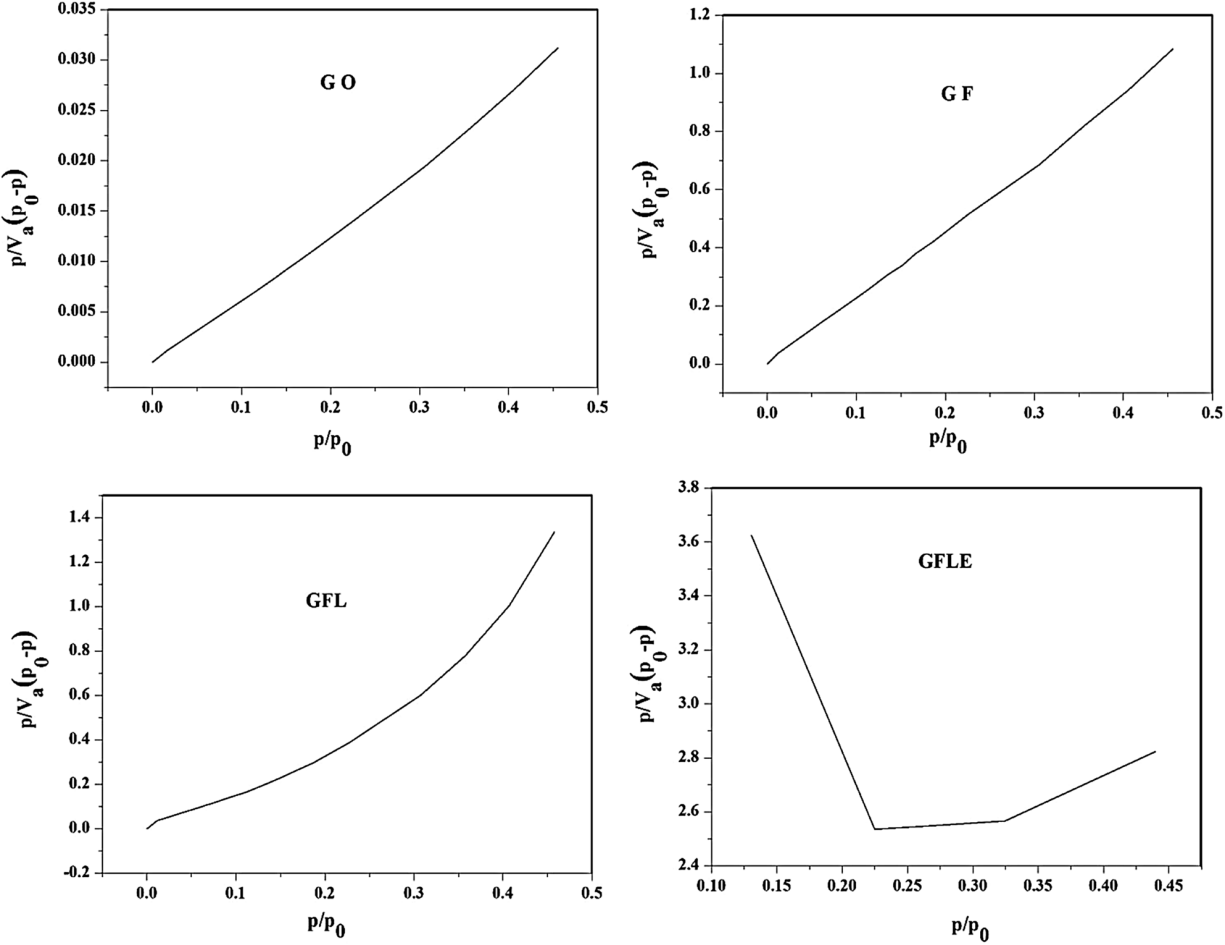
BET curves of GO, GF, GFL and GFLE.

The specific surface area of GO, magnetite GO (GF), magnetite graphene oxide/Lauric acid (GFL) and GFLE measured by the Brunauer–Emmett– Teller (BET) technique is exhibited in Fig. 5 and Table 3. Generally, Surface area of GFLE (3.2897 m^2^ g^−1^), GFL (1.538 m^2^ g^−1^) and GF (1.8474 m^2^ g^−1^) were lower than that of GO (63.647 m^2^ g^−1^ s) due to the high density Fe_3_O_4_, Lauric acid and ethylenediaminetetraacetic acid on the surface graphene oxide.

**Table 3:** BET experimental results of samples.

Sample	Monolayer adsorption volume V_m_ (cm^3^ g^−1^)	BET surface area S_BET_ (m^2^ g^−1^)	Total Pore volume (cm^3^ g^−1^)	Average porediameter D_BET_ (nm)
GO	14.623	63.647	0.2685	16.877
GF	0.4244	1.8474	0.0097314	21.071
GFL	0.3534	1.538	0.030841	80.213
GFLE	0.7558	3.2897	0.0036956	4.4935

**Figure 6:**
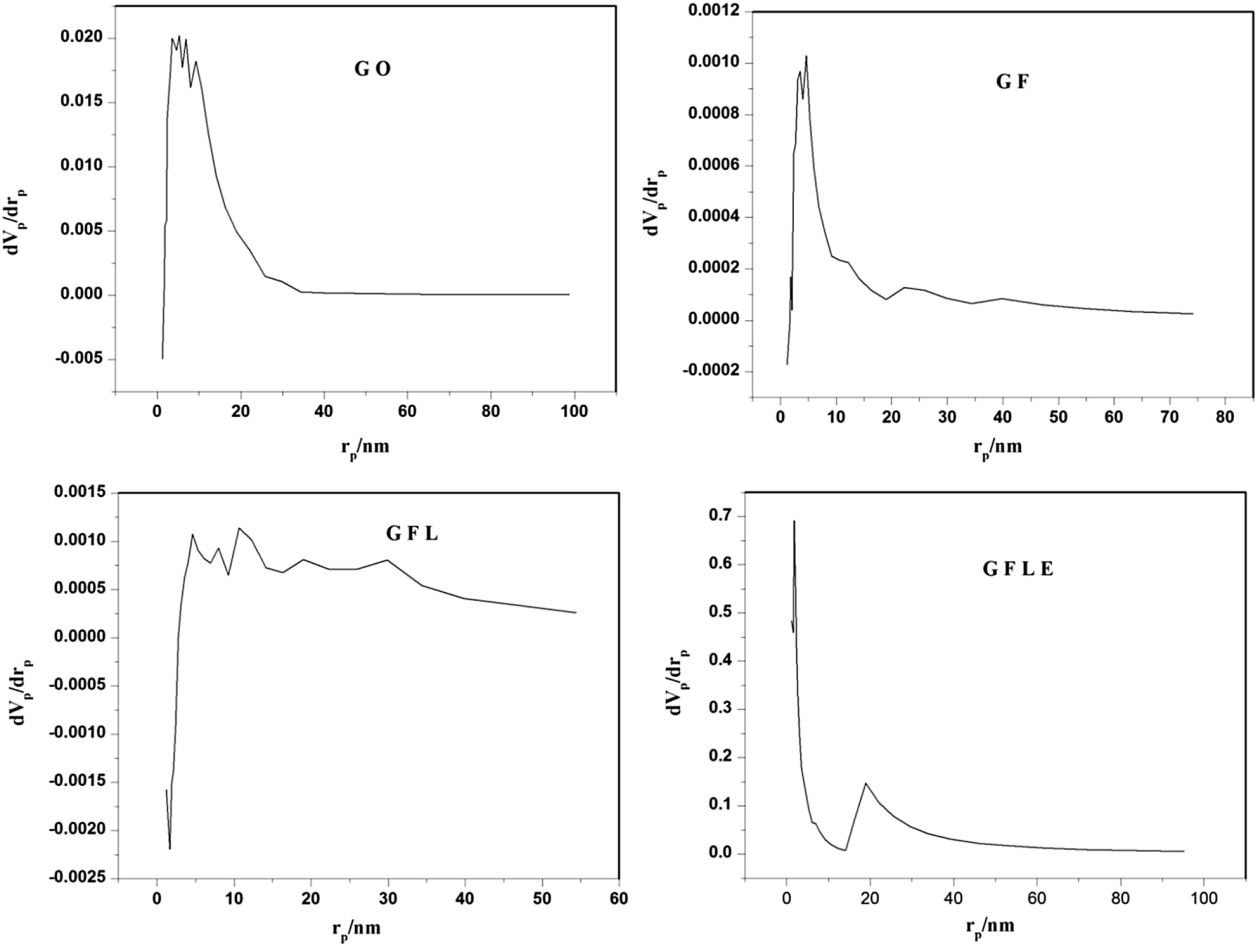
Pore size distribution graphs of GO, GF, GFL and GFLE.

The Barret–Joyner–Halenda (BJH) pore size distribution diagrams of samples are shown in Fig. 6. For all samples studied, the resulting pore size distributions have the form of narrow and asymmetrical peak. These curves shown peaks at 5.29 nm, 4.63 nm, 10.64 nm and 1.85 nm that peaks related to GO, GF, GFL and GFLE, respectively. This means that uniform cylindrical mesopores are formed in samples.

**Figure 7:**
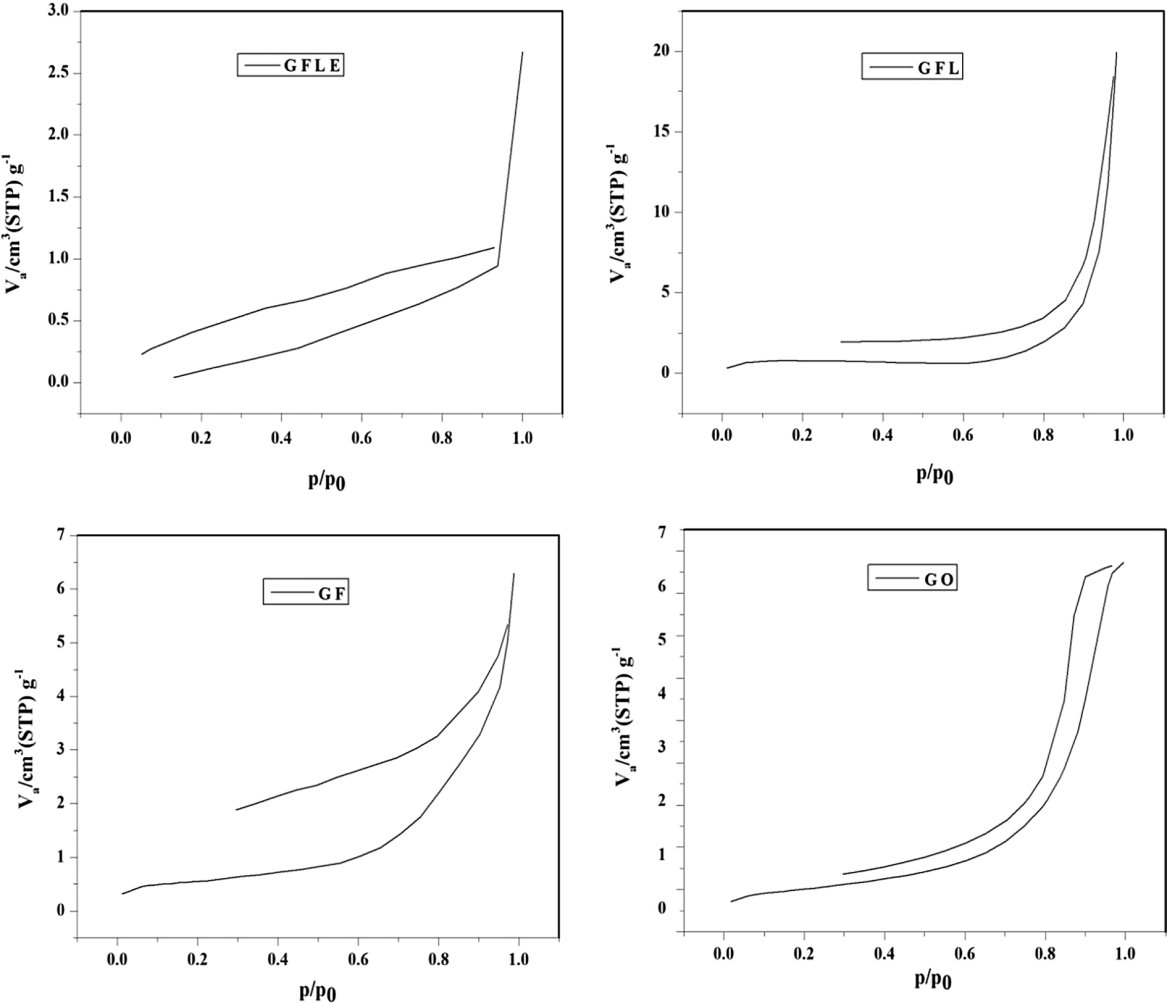
N_2_ adsorption/desorption isotherms of the samples.

The nitrogen adsorption–desorption of the modified nanoporous GFLE samples is presented in Fig. 7. The GFLE pore size distributions were fundamentally the different as before with the graphene oxide surface modification with Fe_3_O_4_, Lauric acid and ethylenediaminetetraacetic acid. However, the surface areas were very different; decreasing with the surface modification with Fe_3_O_4_, Lauric acid and then slightly raising with the surface correction with ethylenediaminetetraacetic acid (Table 4). Each shape of the isotherm showed a distinct hysteresis loop can be employed to qualitatively predict the kinds of pores being in the adsorbent^27^. In Fig. 7 the nitrogen adsorption–desorption of the modified nanoporous samples are shown, which this phenomenon is related with capillary condensation in mesopores or macropores. Pores within porous materials are classified as micropores (< 2 nm), mesopores (2–50 nm), and macropores (> 50 nm), according to IUPAC classification^27^, there for the pore diameter for GO, GF, GFL and GFLE were mesopores (Table 4). The nitrogen adsorption–desorption isotherms of the GO, GF, GFL and GFLE possess IV-type which represents mesoporous structures that these graphs are showed in Fig. 7. Type IV illustrate mono-and multilayer sorption plus capillary condensation^28^. The graphs of hysteresis loops have been used with specific pore structures^28^. In addition, the made hysteresis loops are H1 type (GO), H4 type (GF), H3 type (GFL) and H1 type (GFLE). The results confirms which the porous nearly monotonic spheres in fairly regular and hence to have narrow distributions of pore size for GO and GFLE, for GF that H4 type associated with narrow slit-like pores and the GFL hysteresis loop (H3 type) showed masses of plate-like particles giving rise to slit-shaped pores^28^.

**Table 4:** BJH experimental results of samples.

Sample	Cumulative pore volume of pores V_BJH_ (cm^3^ g^−1^)	Cumulative surface area of pores S_BJH_ (m^2^ g^−1^)	Average pore diameter d_BJH_ (nm)
GO	0.2628	66.719	5.29
GF	0.0096071	2.2854	4.63
GFL	0.030013	1.0094	10.64
GFLE	0.0041492	1.5953	1.85

### EDS results

**Figure 8:**
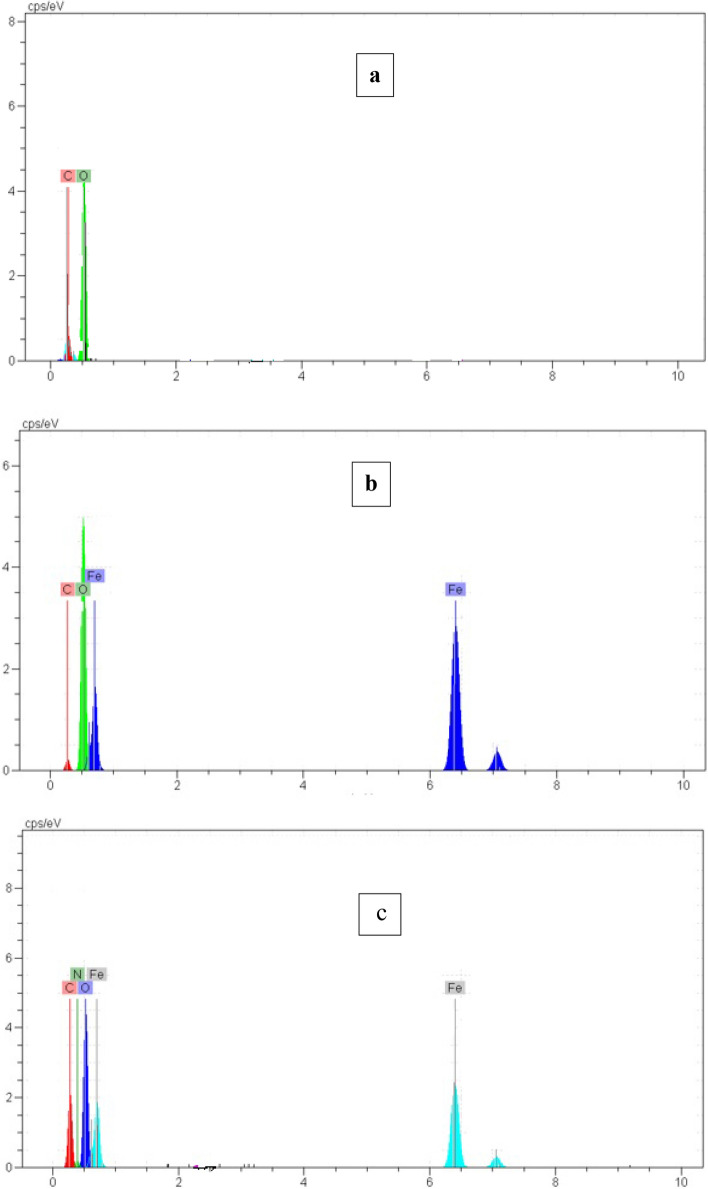
EDX analysis of (**a**) GO, (**b**) GF and (**C**) GFLE nanocomposite.

Figure 8 depicts EDX analysis of GO, GF and GFLE nanocomposite. In the Fig. 8a, GO is combined of O and C. For GF exposed the existence of C, O and Fe elements in the Fig. 8b. Also, EDX spectrum of GFLE is observed in Fig. 8c including Fe, O, C and N.

### RSM methodology for optimization of Cu^2+^ uptake

The responses of CCD analysis for investigating the magnitude of four independent factors along with the predicted mean and obtained answers are seen in Table 5. The quadratic model equation assigning the experimental relationship between residual concentrations (Y) and checked variables were taken in the coded unit and obtained as:

19 $$Y_{{{\text{Re}} sponse}} = 228.98 + 10.52X_{1} + 88.65X_{2} + 0.10X_{3} - 5.31X_{4} + 8.91X_{1} X_{2} - 4.54X_{1}^{2} + 5.15X_{2}^{2} - 4.85X_{4}^{2}$$

**Table 5:** Empirical design based on CCD applied in this paper.

Source	Sum of squares	df	Mean square	F value	*p* value ^a^
Model	1.954E+005	8	24,425.03	222.48	< 0.0001
X_1_	2656.51	1	2656.51	24.20	< 0.0001
X_2_	1.886E+005	1	1.886E+005	1717.88	< 0.0001
X_3_	0.26	1	0.26	2.372E−003	0.9616
X_4_	677.34	1	677.34	6.17	0.0215
X_1_X_2_	1269.14	1	1269.14	11.56	0.0027
X_1_^2^	576.72	1	576.72	5.25	0.0323
X_2_^2^	742.37	1	742.37	6.76	0.0167
X_4_^2^	658.87	1	658.87	6.00	0.0231
Residual	2305.45	21	109.78		
Lack of fit	2303.68	16	143.98	407.11	< 0.0001
Pure error	1.77	5	0.35		

^a^Y_Obs_ = Observed magnitudes of the secondary concentration of Cu (II) (mg L^−1^), Y_Pre_ = Predicted values (mg L^−1^).

**Figure 9:**
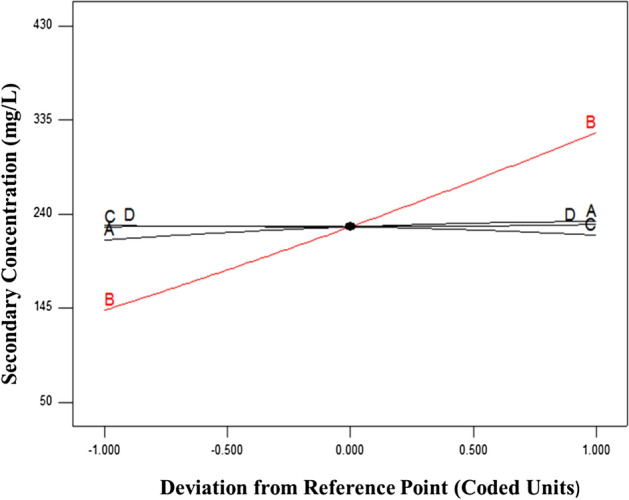
Perturbation curves displaying the influence of process variables on pH (**A**), C_0 Cu_^2+^ (**B**), T (**C**) and t (**D**) on the secondary concentration of Cu^2+^ ions.

In the ANOVA table (Table 6), the F-value (222.48) with a minimum possibility magnitude (*p* < 0.0001) confirmed a great importance for the regression model. The goodness of the model fit was also tested by the multiplex correlation coefficients (R^2^). It can be seen, the magnitude of predicted coefficient (pred. R^2^ = 0.9560) is in equitable compliance with the value of the adjusted coefficient (adj. R^2^ = 0.9839), the indicating great correlation between the seen and the predicted magnitude. Furthermore, the smaller magnitude of the coefficient of variance (CV = 4.64%) shows the significant degree of precision and reliability of the accomplished analyses. Considering the output of the ANOVA table (Table 6) indicated that the quadratic model is statistically important for the prediction of residual concentration. The perturbation plot indicates the results of all the operating parameters at a particular point in the design space. In Fig. 9, the secondary concentration rises by increasing the C_0 Cu_^2+^. The increase of initial ions copper concentration (C_0 Cu_^2+^) elevates the number of interaction between Cu^2+^ ions and GFLE. This behavior because of an increment in the effective driving force (concentration gradient) copper ion concentrations on the cell surface and in the bulk solution, which facilitates sorption. As presented in Fig. 9, pH has minimum impact on the secondary concentration Cu^2+^ ions, the solution with the decrease of pH was not suitable for the freedom of H^+^ from EDTA, and low pH, the coordination of M^2+^could be fundamentally limited. Studying this point, the decrease sorption yield of M^2+^ would be achieved at lower pH. Furthermore, increase pH of the solution was also a disadvantage situation for coordination of M^2+^, that was because of that secondary reaction products of M^2+^ would be afforded, including MOH^+^ and M(OH)_2_ This seriously impacted the uptake performance. Figure 9 displays T and t have least impacts statistically on the secondary concentration Cu^2+^ ions.

**Table 6:** Analysis of variance (ANOVA) for response surface quadratic model of Cu(II) elimination using GFLE nanocomposite.

Std. order	Coded variables								Response^a^	
	X_1_		X_2_		X_3_		X_4_		Y_Obs_	Y_Per_
1	2	(− 1)	170	(− 1)	67.50	(− 1)	30	(− 1)	150	138.71
2	4	(+ 1)	170	(− 1)	67.50	(− 1)	30	(− 1)	150	141.94
3	2	(− 1)	390	(+ 1)	67.50	(− 1)	30	(− 1)	305	298.19
4	4	(+ 1)	390	(+ 1)	67.50	(− 1)	30	(− 1)	342.5	337.04
5	2	(− 1)	170	(− 1)	67.50	(− 1)	50	(+ 1)	132.5	129.33
6	4	(+ 1)	170	(− 1)	67.50	(− 1)	50	(+ 1)	142.5	132.56
7	2	(− 1)	390	(+ 1)	67.50	(− 1)	50	(+ 1)	300	228.81
8	4	(+ 1)	390	(+ 1)	67.50	(− 1)	50	(+ 1)	337.5	327.67
9	2	(− 1)	170	(− 1)	142.5	(+ 1)	30	(− 1)	152.5	138.92
10	4	(+ 1)	170	(− 1)	142.5	(+ 1)	30	(− 1)	160	142.15
11	2	(− 1)	390	(+ 1)	142.5	(+ 1)	30	(− 1)	302.5	298.40
12	4	(+ 1)	390	(+ 1)	142.5	(+ 1)	30	(− 1)	342.5	337.25
13	2	(− 1)	170	(− 1)	142.5	(+ 1)	50	(+ 1)	127.5	129.54
14	4	(+ 1)	170	(− 1)	142.5	(+ 1)	50	(+ 1)	137.5	132.77
15	2	(− 1)	390	(+ 1)	142.5	(+ 1)	50	(+ 1)	305	289.02
16	4	(+ 1)	390	(+ 1)	142.5	(+ 1)	50	(+ 1)	332.5	327.87
17	1	(− 2)	280	(0)	105	(0)	40	(0)	185	190.50
18	5	(+ 2)	280	(0)	105	(0)	40	(0)	212.5	232.58
19	3	(0)	60	(− 2)	105	(0)	40	(0)	52.5	73
20	3	(0)	500	(+ 2)	105	(0)	40	(0)	422.5	427.58
21	3	(0)	280	(0)	105	(0)	20	(− 2)	192.5	215.92
22	3	(0)	280	(0)	105	(0)	60	(+ 2)	195	197.17
23	3	(0)	280	(0)	30	(− 2)	40	(0)	220	228.95
24	3	(0)	280	(0)	180	(+ 2)	40	(0)	225	229.37
25	3	(0)	280	(0)	105	(0)	40	(0)	226.3	229.16
26	3	(0)	280	(0)	105	(0)	40	(0)	227.5	229.16
27	3	(0)	280	(0)	105	(0)	40	(0)	227.5	229.16
28	3	(0)	280	(0)	105	(0)	40	(0)	227.5	229.16
29	3	(0)	280	(0)	105	(0)	40	(0)	227.5	229.16
30	3	(0)	280	(0)	105	(0)	40	(0)	226.4	229.16

R^2^ = 0.9883; Adj R^2^ = 0.9839, Pred R^2^ = 0.9560, Coefficient of variance = 4.64%.

^a^ *p* < 0.01, highly significant; 0.01 < *p* < 0.05 significant; *p* > 0.05, not significant.

**Figure 10:**
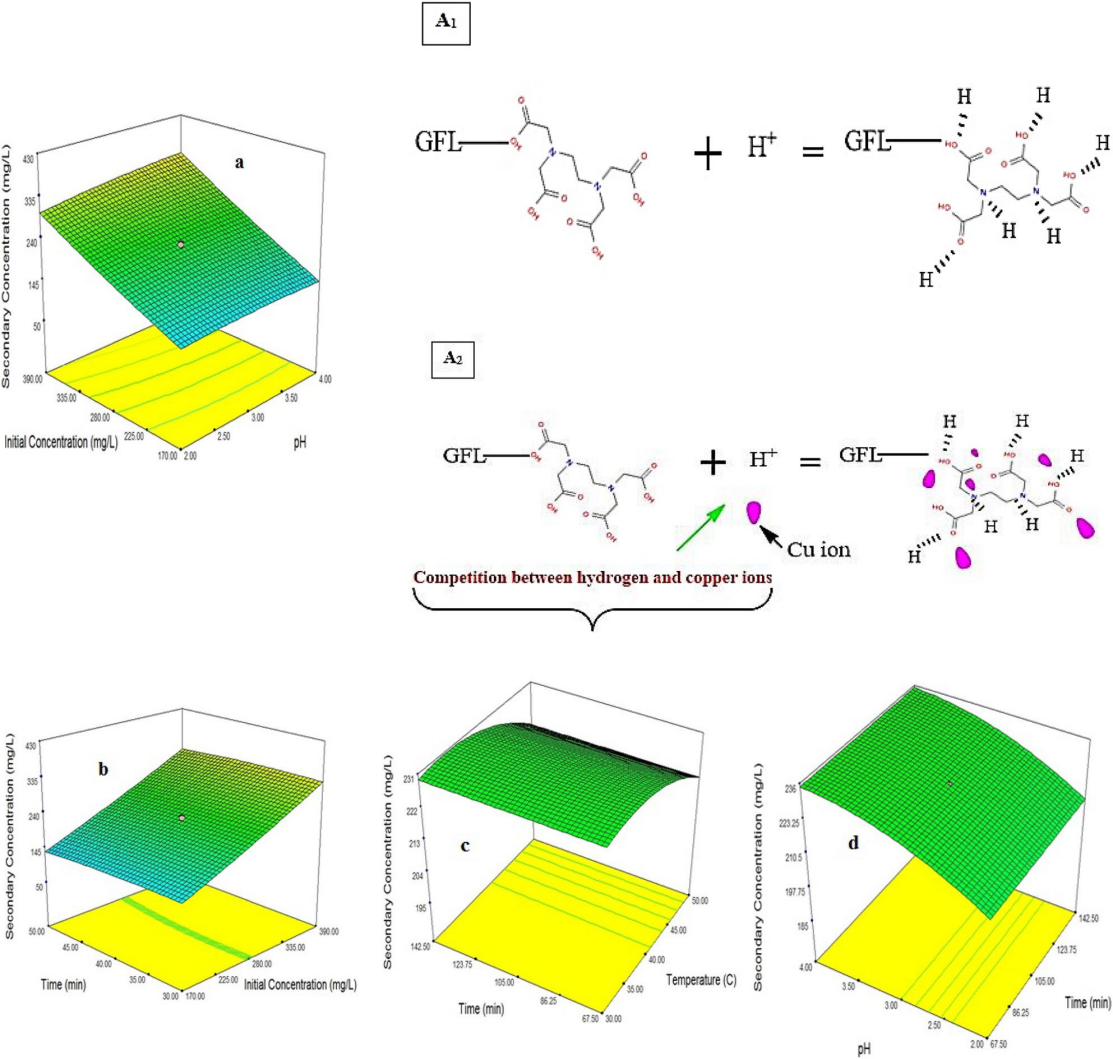
3D response surface graphs indicating the impacts of mutual interactions between two independent variables A_1_ and A_2_ process Cu^2+^ adsorption on GFLE.

Figure 10a demonstrations the interaction result of pH and concentration of copper solution on the secondary concentration of copper in the adsorption process. According to Fig. 10a and Eq. (18) pH (+ 10.52X_1_) and concentration (+ 88.65X_2_) have been the minimum and maximum impact on the adsorption, respectively. The Cu^2+^ adsorption at pH = 1 could be described with the following Eqs. (20), (21) and Fig. 10A1,A2, which depicted process Cu^2+^ adsorption took place at the solid-solution boundary of GFLE adsorbent^9^:

20 $$\left\{ \begin{gathered} GFLE(COOH) + H^{ + } = GFLE(COOH_{2}^{ + } ) \hfill \\ GFLE(COOH) + Cu^{2 + } = GFLE(COOH)Cu^{2 + } \hfill \\ \end{gathered} \right\}$$

21 $$\left\{ \begin{gathered} GFLE(NH_{{}} ) + H^{ + } = GFLE(NH_{2}^{ + } ) \hfill \\ GFLE(NH) + Cu^{2 + } = GFLE(NH)Cu^{2 + } \hfill \\ \end{gathered} \right\}$$

The adsorption process on GFLE enhanced with the increment of C_0 Cu_^2+^ in the range of 60–500 mg L^−1^ while pH had minimum influence on the adsorption process. Therefore, at higher concentration of metal ions, the mass conduct driving force and the number of collisions between Cu^2+^ ions and the adsorbent increased that ultimately raised the sorption mechanism^7^.

The relevance between C_0 Cu_^2+^ and time is presented in Fig. 10b. In Eq. (19) the show, which time had the minimum (+ 0.01X_3_) effective parameter on the adsorption yield. An effect of the initial Cu^2+^ concentration in Fig. 10b was similar to Fig. 10a. As shown in Fig. 10c, the temperature 40 °C had maximum adsorption yield and time was less effective. The result displayed that sorption of Cu^2+^ ions rises with increasing temperature in 40 °C, next rise in temperature (more 40 °C) cause decrease in the adsorption process that it can be related to either the loss of active binding sites in the absorbent or increasing tendency to desorbed Cu^2+^ ions from the interface to the solution because with raising T, the attractive forces between absorbent surface and metal ions are weakened and the sorption decreases^20^. Figure 10d displays the interaction effects of initial solution pH and t on Cu^2+^ uptake, according to Eq. (19) time (+ 0.01X_3_) has had the least impact then pH (+ 10.52X_1_) on the adsorption yield. The increasing Cu^2+^ initial concentration accelerated the diffusion of Cu^2+^ ions from solution to the active sites on the beads of adsorbent because of the rise in concentration gradient driving force, but it is apparent which the adsorption rate achieved at lower initial Cu^2+^ concentrations is faster compared to higher concentrations. With increasing initial Cu^2+^ ions concentration, aggregation phenomenon increased which caused the secondary Cu^2+^ concentration to increase^30^. The adsorption yield increased with the decrease of initial solution pH, and an increase in contact time only slightly affected the uptake mechanism. As the temperature rises, the secondary concentration of Cu^2+^ ions increases while it decreases with time, because higher temperatures render more metal ions capable to dominate the activation energy of the reaction, increases the diffusion which leads to more transformation^31^. Upper a definite temperature, the ligands are instable, that caused in the decrease conversion. The optimum status for the least secondary concentration of copper or the higher sorption (185 mg L^−1^) were obtained to be as follows: pH = 1, the initial Cu^2+^ concentration of 280 mg L^−1^, the T of 40 °C and t of 105 min (Table 7).

**Table 7:** The proposed levels of parameters studied to minimize the secondary concentration of Cu (II) and validation of laboratory experiments.

Factor	The initial concentration of copper (mg L^−1^)	pH	Time (min)	Temperature (°C)	The secondary concentration of copper (mg L^−1^)
Model projections	280	1	105	40	193.389
Model validation	280	1	105	40	185

### Interpretation of residual diagrams

**Figure 11:**
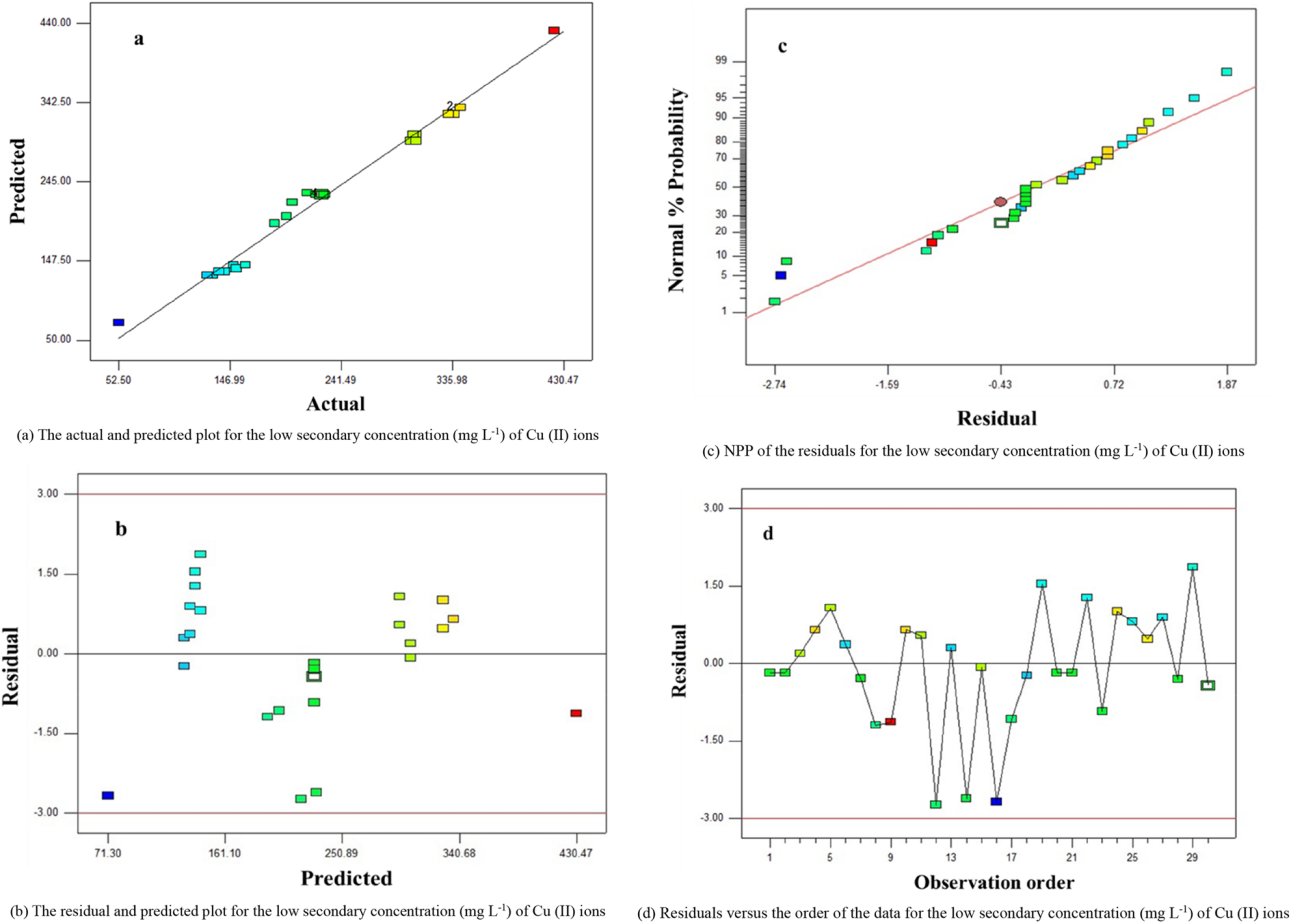
Residual graphs (**a**) the actual and predicted plot, (**b**) the residual and predicted plot, (**c**) normal probability plot (**d**) residuals vs. the order of the data.

The normal probability plot (NPP) is a graphical method for investigating that the result from the empirical is approximately normally dispersed. If the points on the diagram fall justly nearly a straight line, therefore, the data are normally dispersed. The residual is the different between the experimental results and the predicted results (or fitted results) from the regression analysis^30^. Based on Eqs. 19, the observed and predicted plot for the minimum secondary concentration (mg L^−1^) of Cu (II) ions using GFLE is displayed in Fig. 11a, which displayed a well agreement between observed data and predicted response. Figure 11b also indicates graph the residuals against the anticipated response, that the residuals are scattered accidentally about zero i.e. the errors have a constant variance. Figure 11c shows the normal probability graph of residual values and the empirical points were reasonably aligned showing normal distribution. Figure 11d exhibit graphs the residuals in the order of the relating descriptions. The residuals give the impression to be randomly scattered about zero and all other points were observed to fall in the range of + 3 to − 3 except points + 3 and − 3.

### Optimization of adsorption process and model validation

**Figure 12:**
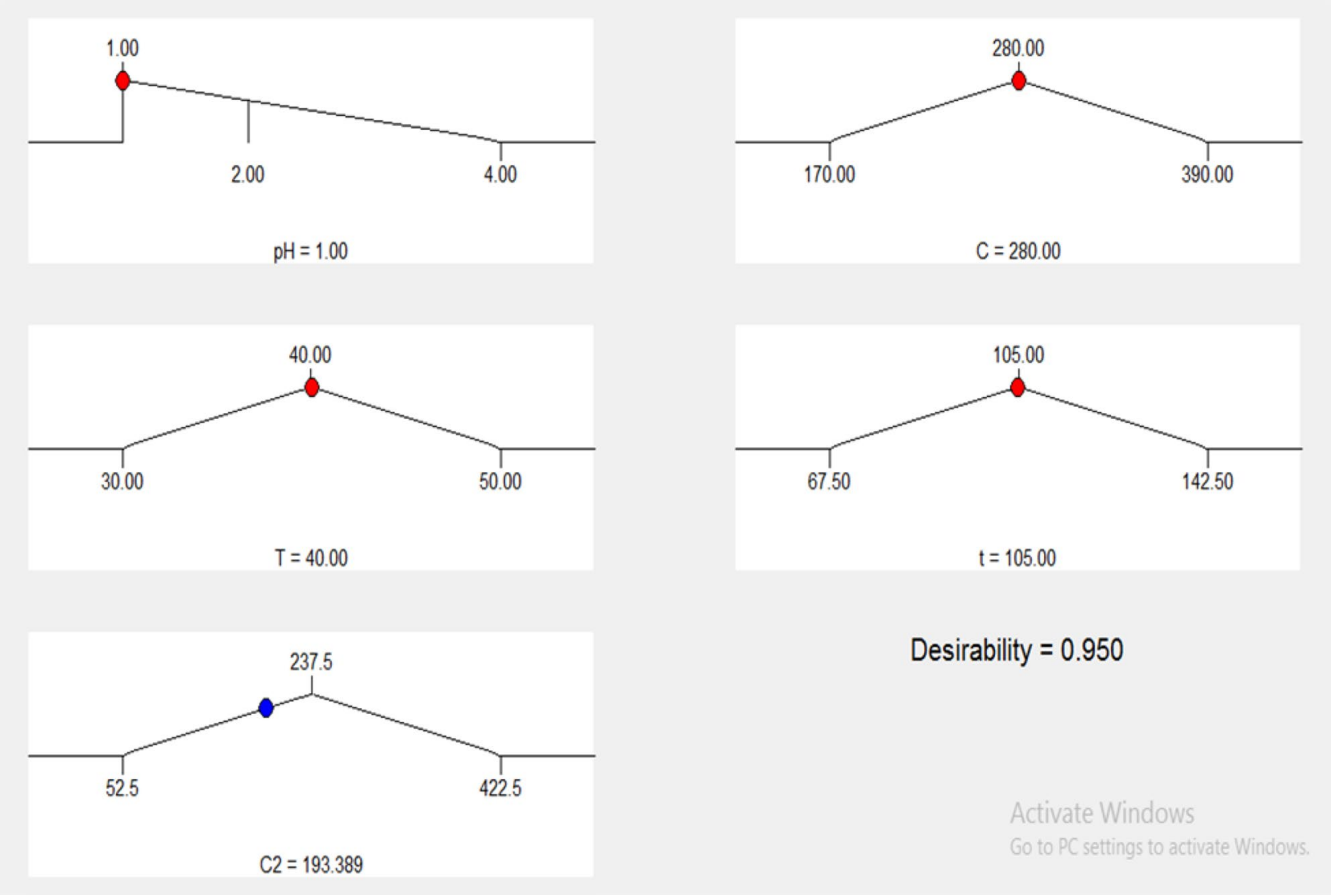
Desirability ramp for optimization.

Optimization of the process factors to increase the uptake of Cu^2+^ ions on GFLE was achieved using the quadratic model. Optimum condition selected was considered using Design Expert Software that is exhibited in Fig. 12. It can be seen that the higher sorption capacity was 95 mg g^−1^ at an initial copper concentration of 280 mg L^−1^, pH = 1, the temperature of 40 °C and time of 105 min. To check the credibility of the model, three verification tests were organized at the anticipated optimal situations to higher uptake capacity, which the average of three extra adsorption experiments were described in Table 7. The assenting analysis displayed the minimum secondary concentration of copper by GFLE 185 mg L^−1^ (or adsorption capacity = 95 mg g^−1^) under optimum situations compared with the minimum secondary concentration of 193.389 mg L^−1^ achieved via the model. This illustrates, that model developed by RSM was highly suitable and accuracy for the copper removal from aqueous solutions by GFLE nanocomposite.

### Adsorption kinetics

**Figure 13:**
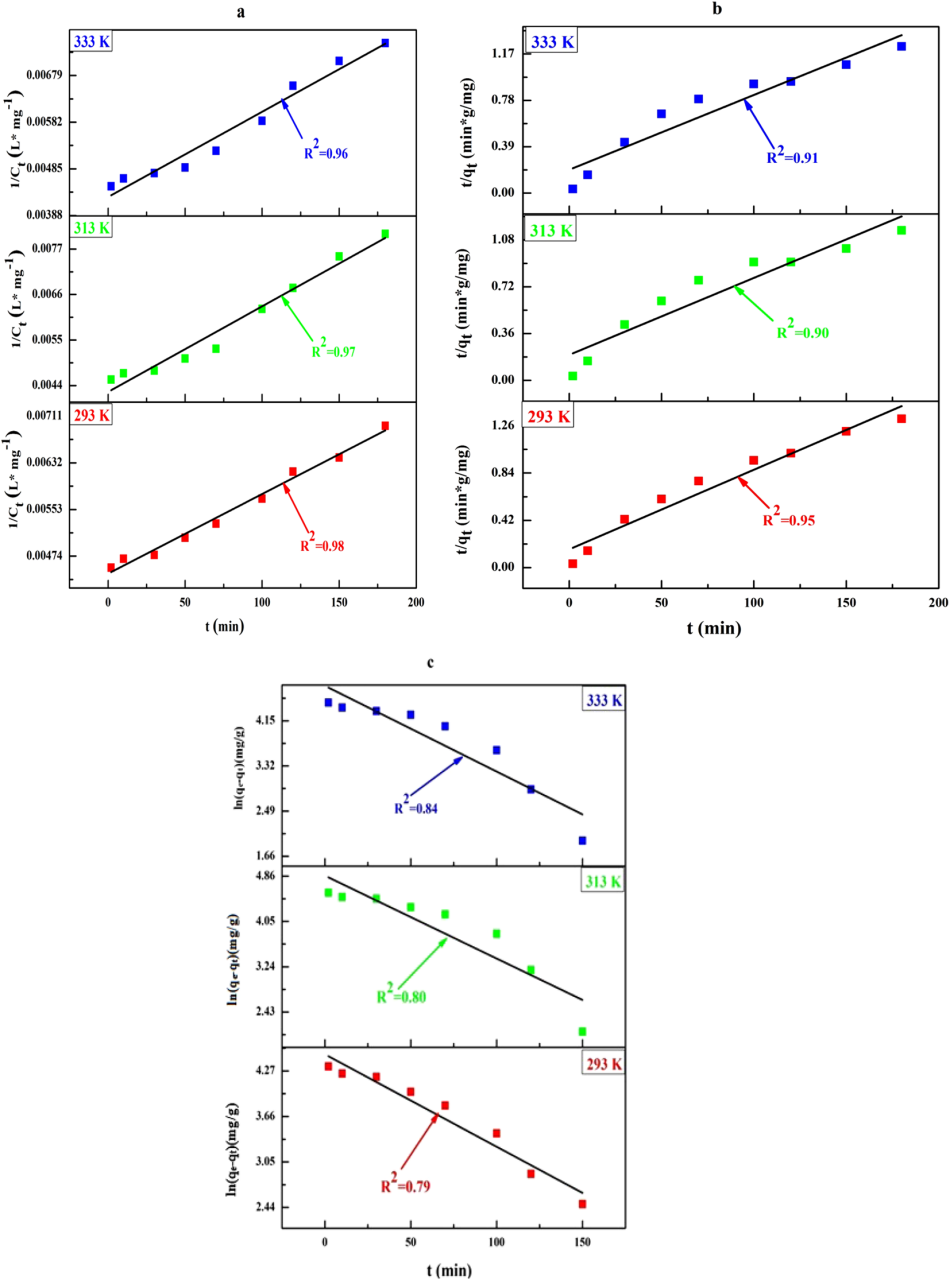
(**a**) Second-order and (**b**) pseudo-second-order (**c**) pseudo-first-order kinetics for adsorption of the Cu (II) ions onto GFLE at 293, 313 and 333 K.

The uptake of Cu^2+^ ions from aqueous solution on GFLE as a function of t is displayed in Fig. 13 and the data of kinetic model fittings are reported in Table 8. The responses of the linear fitting of the empirical data with the second-order kinetic model presented better correlation coefficient (R^2^) (closer to unity appraised to the pseudo first-order and pseudo-second-order models) that indicated the kinetics of Cu^2+^ ions adsorption by GFLE is described well through second-order model that demonstrates that the rate-limiting step can be ion exchange reactions between adsorbent and adsorbate^15^.

**Table 8:** Kinetic variables of Cu (II) ions adsorption on GFLE at 293, 313 and 333^o^K.

Ordermodel	Parameters	Temperature (°K)		
		293	313	333
Second-order model	C_0_ Cal. (mg g^−1^)	227	238	232
	C_e_ Exp. (mg g^−1^)	280	280	280
	K_2_ (min^−1^)	1E−05	2E−05	2E−05
	R^2^	0.98	0.97	0.96
Pseudo-second-order model	qe Calc. (mg g^−1^)	140.84	169.49	158.73
	qe Exp. (mg g-^−1^)	136	156	146
	Kʹ_2_ (g mg^−1^ min^−1^)	3.1E−04	1.7E−04	2.02E−04
	R^2^	0.95	0.90	0.91
	$\chi^{2}$	1.2	10.49	9.98
Pseudo-first-order model	qe Calc. (mg g^−1^)	123	178	300
	qe Exp. (mg g^−1^)	136	156	146
	K_1_ (g mg^−1^ min^–1^)	0.0197	0.0219	0.022
	R^2^	0.79	0.80	0.84
	$\chi^{2}$	10.99	24.82	1299

**Figure 14:**
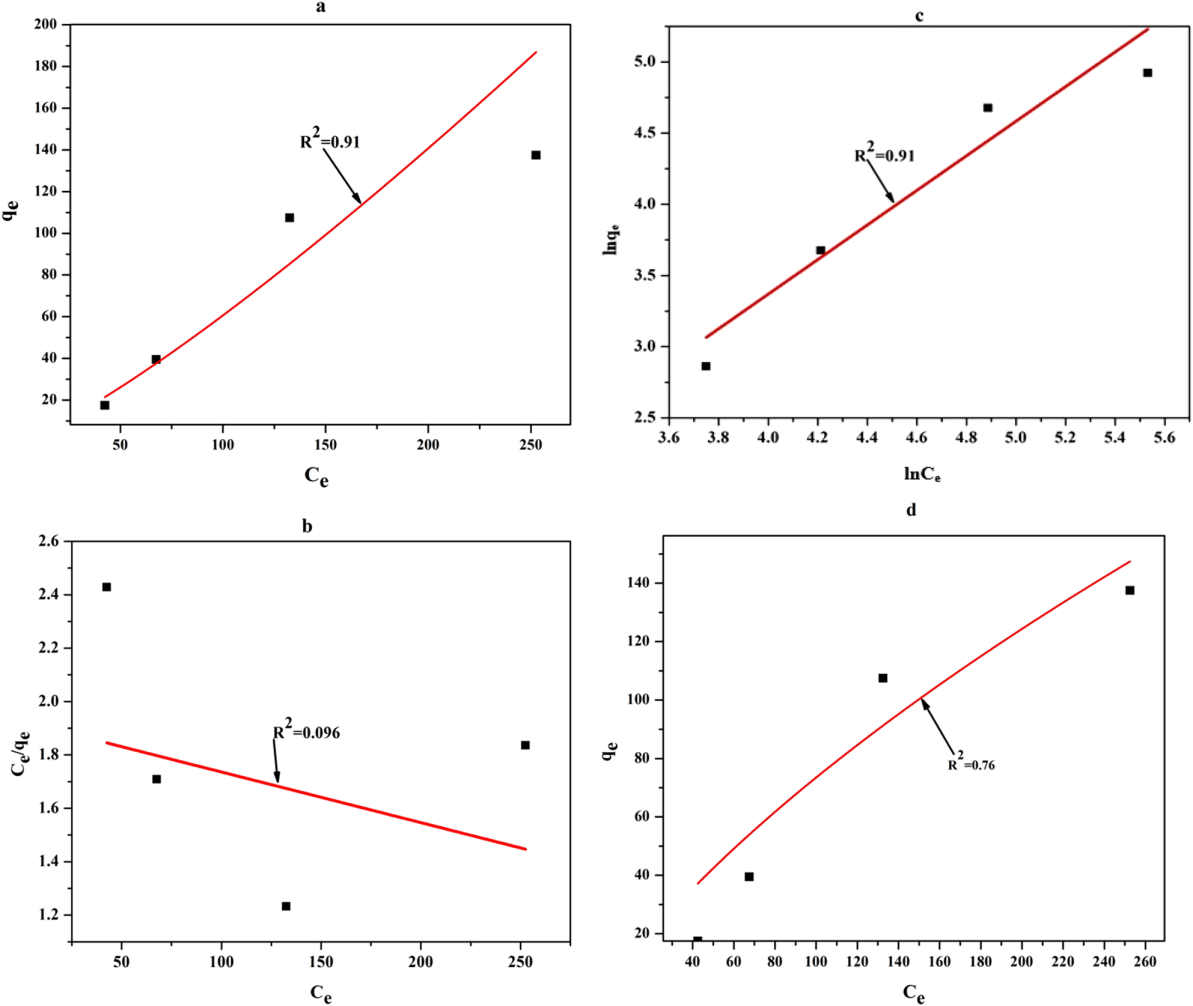
Plot of isotherms for adsorption of Cu^2+^ ions on GFLE.

The equilibrium data were also fitted to the Freundlich, Langmuir, Temkin, and Redlich–Peterson isotherms models with the obtained parameters of indicated in Fig. 14 and Table 11. evaluating the R^2^ and $\chi^{2}$ value of all the isotherms in Table 9, it can be observed that both Freundlich and Temkin adsorption isotherms best fit the empirical equilibrium data. Therefore, it can be resulted that, the uptake is based on the multilayer formation of Cu^2+^ ions adsorbed on the heterogeneous surface of the adsorbent. ‘n’ value for Cu^2+^ ions sorption (1.2 > 1) presented that the adsorption was favorable.

**Table 9:** Freundlich, Langmuir and Temkin parameters for the sorption of Cu (II) ions on GFLE.

Ordermodel	Parameters	
	n	1.2
Freundlich isotherm model	K_f_ (mg g^−1^)	0.225
	R^2^	0.91
	$\chi^{2}$	33.62
Langmuir isotherm model	q_max_. (mg g^−1^)	526
	b	9.87E–004
	R^2^	0.096
	$\chi^{2}$	4390.7
Temkin isotherm model	A (Lg^−1^)	2.9E–112
	b_t_ ( KJ mol^−1^)	0.034
	R^2^	0.91
	$\chi^{2}$	3.9
Redlich–Peterson	$K_{R}$	32.56
	$a_{R}$	34.05
	$\beta$	0.99
	R^2^	0.76
	$\chi^{2}$	541.94

The obtained thermodynamic parameters ($\Delta G^{ \circ }$, $\Delta H^{ \circ }$ and $\Delta S^{ \circ }$) are presented in Table 10. The increase in $\Delta G^{ \circ }$ value at 313 K and the decrease in the magnitude of $\Delta G^{ \circ }$ at 333 K show that the adsorption mechanism is more favorable at 313 K. The negative magnitudes of $\Delta G^{ \circ }$ indicates the possibility of the method and spontaneous nature of Cu^2+^ ions uptake onto GFLE nanocomposite.

**Table 10:** Thermodynamic factors for Cu (II) adsorption onto GFLE nanocomposite.

$T \left( {^\circ K} \right)$	$\Delta G^\circ \left( {\frac{KJ}{{mol}}} \right)$	$\Delta S^\circ \left( \frac{J}{molK} \right)$	$\Delta H^\circ \left( {\frac{KJ}{{mol}}} \right)$
293	− 0.51		
313	− 0.65	− 2.660	0.242
333	− 0.60		

The amounts of $\Delta G^{ \circ }$ (− 0.51 to − 0.60) for the adsorption of Cu^2+^ in the proposed nanoadsorbent are in the range of physical uptake^18^.

The positive magnitude of $\Delta H^{ \circ }$ verifies the endothermic nature of Cu^2+^ sorption process that is further stabilized through the decrease in Cu^2+^ sorption with the rise in temperature. The positive magnitude of $\Delta S^{ \circ }$ implies the affinity of the GFLE for copper as well as increase of randomness at solid–solution boundary through metal ion uptake.

### Determination of activation energy

**Figure 15:**
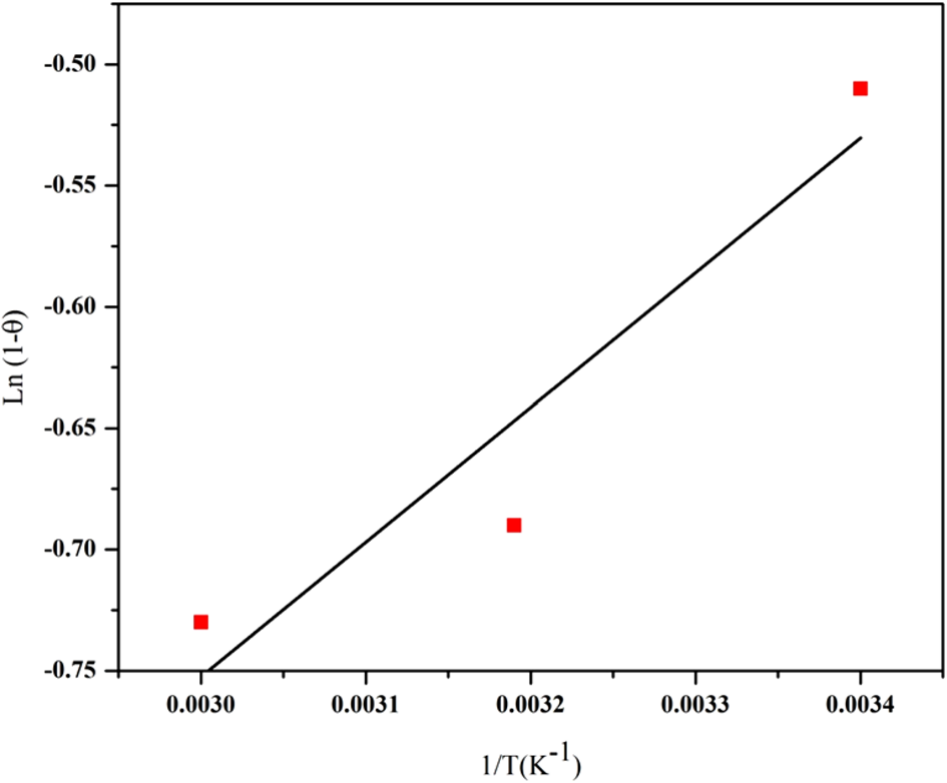
Graph of Ln (1 − θ) versus 1/T for uptake of Cu^2+^ ions on GFLE.

The positive magnitude of E_a_ in Fig. 15 reveals that a higher temperature favors copper adsorption on GFLE nanocomposite and the sorption process is endothermic in nature. Activation energy magnitude is usually employed as the basis for differentiating the nature of uptake, whether it is physical or chemical^17^. In this regard, if the value of Ea is between 8.4 and 83.7 kJ mol^−1^, therefore the uptake is formed using strong forces indicating chemical adsorption whenever activation energies of E_a_ < 8 kJ mol^−1^ relate to physical nature of the uptake mechanism^8,19^. The E_a_ magnitude for the sorption of Cu^2+^ ions onto magnetic nanoadsorbent was determined to be 4.61 kJ mol^−1^ (R^2^ = 0.89) offering which physisorption was the major process of sorption. For S* > 1 there is no interplay between adsorbent and adsorbate, and so no uptake happens, S* = 1 is assigned to the probability that physisorption and chemisorption coexist, S* = 0 related to the influence of the chemisorption process. Desirable grafting of adsorbate to adsorbent happens by physisorption process when S* lies in the range 0 < S* < 1^17^. The magnitude of sticking probability was calculated as 0.0837 which corresponds to the physical nature of adsorption mechanism.

### Desorption study

**Figure 16:**
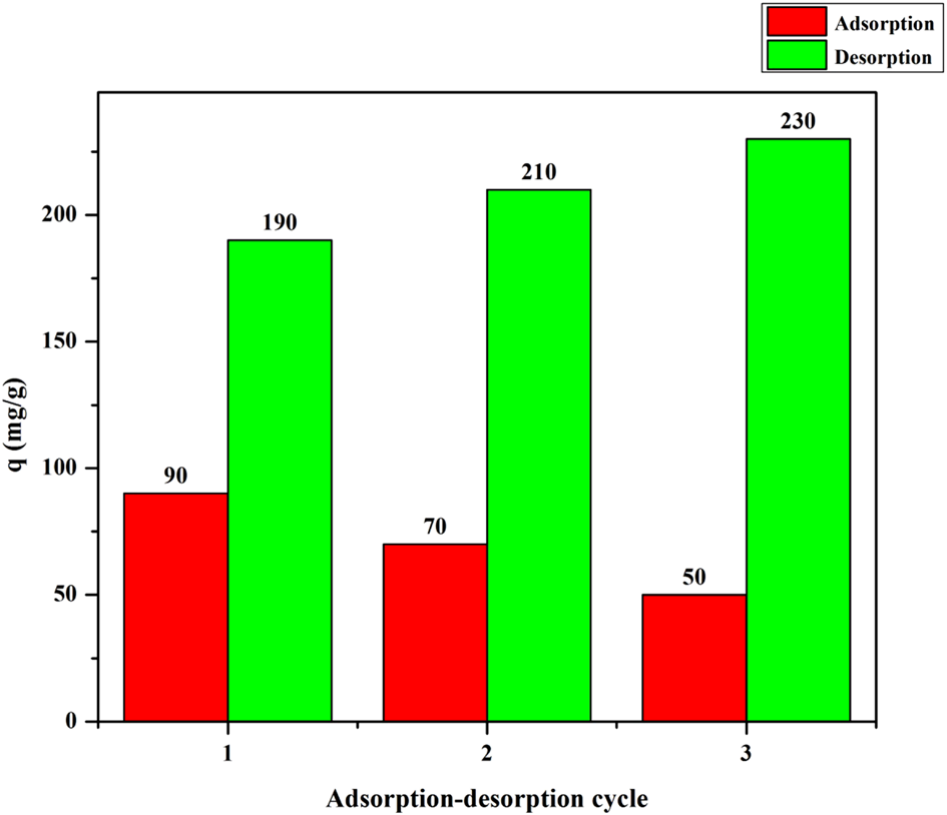
Adsorption/desorption with repeated cycles on GFLE; Initial concentration 280 mg L^−1^ Cu (II), pH = 1, time 105 min and temperature 313 K.

The reusability of a benefit adsorbent is significant in economic development because the repeated availability is the key factor to evaluate the applicability of an adsorbent. Desorption of Cu^2+^ from GFLE nanoadsorbent was performed using 0.2 M Na_2_EDTA repeated in 3 cycles with the same dose. Figure 16 shows the continuous adsorption– desorption cycles of Cu^2+^ on synthesized nanocomposite in the appointing maximum uptake adsorption–desorption situations. It is clear that sorption of Cu^2+^ reduced slightly from 90 to 50 mg g^−1^ within 3 consecutive cycles. This decrement may be relate to the destroyer influence of the stripping agent and mass loss of the adsorbent in desorption process. Furthermore, the resident of Cu^2+^ ions on GFLE nanocomposite (irreversible binding) caused in a low in the number of available sorption sites^21^. Thus, it is obvious that physical sorption must have performed a main character in the uptake of copper ions onto the nanoadsorbents. This evidence displayed that GFLE nanocomposite has remarkable ability for the sorption of Cu^2+^ ions from aqueous solutions.

### Comparison with various adsorbents

The mechanism of Cu^2+^ adsorption onto GFLE nanocomposite has been similar sorption Pb^2+^ onto GFLE^11^. Table 11 demonstrated that adding Fe_3_O_4_ and LA to the surface of GO had no obvious effect on the absorption capacity while it was increased after EDTA groups were added on to GFL surface. It is obvious which the EDTA group can rise the sorption abilities of the Cu^2+^ ions. Functionalized GFL with EDTA as a strong chelating hexadentate ligand that can considerably raise the adsorption potentials of the copper ions in which the coordination interplay between EDTA and Cu^2+^ was one of the causes that effected in the high adsorption capacity. Furthermore, EDTA increases the number of oxygen-containing functional groups on the surface of GO and therefore causes an increment in GFLE adsorption potency for Cu^2+^ deletion^22,23^. Also, in Table 11 a comparison of the different absorbents used to remove copper with the one in this study is presented.

**Table 11:** Comparison of sorption capacities of several adsorbents for Cu (II) ions.

Adsorbent	Q (mg g^−1^)	References
EDTA-mGO	301.2	^22^
Sulfonated magnetic graphene oxide composite	62.73	^7^
Magnetic chitosan/graphene oxide nanocomposites	217.4	^24^
Graphene Oxide functionalized with ethylenediamine triacetic acid	108.7	^23^
Magnetic graphene oxide composite	62.73	^25^
Magnetic Dithiocarbamate Functionalized Reduced Graphene Oxide	113.64	^26^
Graphene oxide	65	This work
GF	65	This work
GFL	65	This work
GFLE	95	This work

### Cost analysis of adsorbents

In the study, an effort has been synthesized to investigate the cost of adsorbent GFLE nanocomposite. The cost analysis for the preparation of 1 g of adsorbent was calculated as 300000R.

### Adsorption mechanism

**Figure 17:**
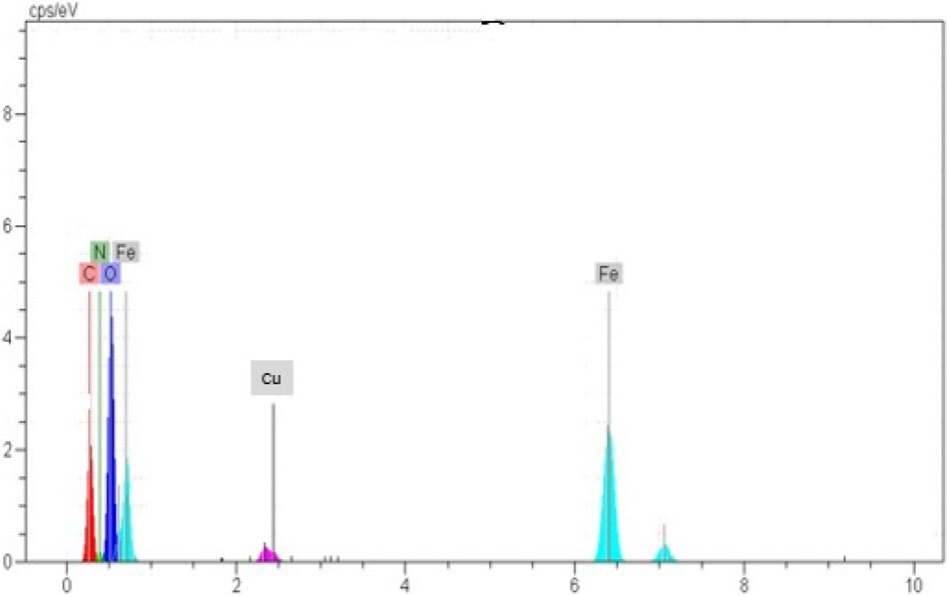
EDX spectrum of GFLE after adsorption Cu (II).

According to the result obtained from kinetic models, adsorption isotherms, thermodynamic and activation energy the adsorption mechanism of Cu (II) on GFLE nanocomposite is ion exchange, endothermic and spontaneous nature. Figure 17 display EDX analysis of GFLE, after the adsorption of Cu (II). Mechanism of copper removal by the GFLE nanocomposite is shown in Eq. (22)^11^:

22 $$(GO/Fe_{3} O_{4} /LA/EDTA - COOH_{2} ) + Cu^{2 + } \to (GO/Fe_{3} O_{4} /LA/EDTA - COO)_{2} Cu + 2H^{ + }$$

## Conclusions

GFLE nanocomposite was made by coprecipitation. The influences of variables include pH, t, C_0 Cu_^2+^, and T for investigating the uptake process of Cu^2+^ ions in a batch adsorption system were evaluated using RSM. Based on the obtained results, the produced nanoadsorbent has the potential to be used as a good adsorbent for eliminating Cu^2+^ ions. Studies of the kinetic models and adsorption isotherms displayed that the adsorption of copper onto GFLE can be modeled using second-order kinetic models and Freundlich isotherm. Thermodynamic studies defined the endothermic and spontaneous nature of the uptake mechanism. Also, the achieved activation energy magnitude was 4.61 kJ mol^−1^ exhibiting which the sorption mechanism is based on physisorption. In research shows that the GFLE nanocomposite could be operated as the low-cost adsorbent for the deletion of Cu^2+^ ions due to quick kinetics, great adsorption capacity, and high regeneration capabilities even after 3 adsorption–desorption cycles. The time and pH had less effect on the sorption capacity compared to other varied parameters including concentration and temperature. In this study, we suggested two new materials (LA and EDTA(, for the made of GFLE by the method of co-precipitation and the superparamagnetic properties of the adsorbent were applied to eliminate copper ions from the aqueous sample.

## References

[1] Liu H (2014). Removal of Cu (II) ions from aqueous solution by activated carbon impregnated with humic acid. J. Front. Environ. Sci. Eng..

[2] Aydın H, Bulut Y, Yerlikay C (2008). Removal of copper (II) from aqueous solution by adsorption onto low-cost adsorbents. J. Environ. Manag..

[3] Khandanlou R (2016). Enhancement of heavy metals sorption via nanocomposites of rice straw and Fe_3_O_4_ nanoparticles using artificial neural network (ANN). J. Ecol. Eng..

[4] Javid N, Malakootian M (2017). Removal of bisphenol A from aqueous solutions by modified-carbonized date pits by ZnO nano-particles. J. Desalin. Water Treat..

[5] Farsi A, Javid N, Malakootian M (2019). Investigation of adsorption efficiency of Cu^2+^ and Zn^2+^ by red soil and activated bentonite from acid copper mine drainage. J. Desalin. Water Treat.

[6] Honarmandrad Z, Javid N, Malakootian M (2020). Efficiency of ozonation process with calcium peroxide in removing heavy metals (Pb, Cu, Zn, Ni, Cd) from aqueous solutions. J. Appl. Sci..

[7] Hu X (2013). Removal of Cu (II) ions from aqueous solution using sulfonated magnetic graphene oxide composite. J. Sep. Purif. Technol..

[8] Zulfiqar Ali S (2011). Simultaneous removal of Pb (II), Cd (II) and Cu (II) from aqueous solutions by adsorption on Triticum aestivum—a green approach. J. Hydrol. Curr. Res..

[9] Yong-Mei H, Man C, Zhong-Bo H (2010). Effective removal of Cu (II) ions from aqueous solution by amino-functionalized magnetic nanoparticles. J. Hazard. Mater..

[10] Mao N (2012). Adsorption performance and mechanism of Cr (VI) using magnetic PS-EDTA resin from micro-polluted waters. J. Chem. Eng..

[11] Danesh N (2015). Fabrication, characterization and physical properties of a novel magnetite graphene oxide/Lauric acid nanoparticles modified by ethylenediaminetetraacetic acid and its applications as an adsorbent for the removal of Pb(II) ions. J. Synth. Met..

[12] Huang Q (2015). A facile and green method for synthesis of reduced graphene oxide/Ag hybrids as efficient surface enhanced Raman scattering platforms. J. Hazard. Mater..

[13] Jonidi Jafari A (2016). Application of mesoporous magnetic carbon composite for reactive dyes removal: Process optimization using response surface methodology. Korean J. Chem. Eng..

[14] Ghorbani F (2008). Application of response surface methodology for optimization of cadmium biosorption in an aqueous solution by Saccharomyces cerevisiae. J. Chem. Eng..

[15] Ho Y (2008). Review of second-order models for adsorption systems. J. Hazard. Mater. B..

[16] Rostamian R, Najafi M, Rafati AA (2011). Synthesis and characterization of thiol-functionalized silica nano hollow sphere as a novel adsorbent for removal of poisonous heavy metal ions from water: Kinetics, isotherms and error analysis. Chem. Eng. J..

[17] Pashai Gatabi, M. Milani Moghaddam, H. & Ghorbani, M. *Nanopart Res J*. Doi: 10.1007/s11051-016-3487-x (2016).

[18] Konicki W (2013). Equilibrium and kinetic studies on acid dye Acid Red 88 adsorption by magnetic ZnFe_2_O_4_ spinel ferrite nanoparticles. J. Colloid Interface Sci..

[19] Ciopec M (2012). Adsorption studies of Cr (III) ions from aqueous solutions by DEHPA impregnated onto Amberlite XAD7 – Factorial design analysis. J. Chem. Eng. Res. Des..

[20] Hasan SH, Srivastava P, Talat M (2009). Biosorption of Pb (II) from water using biomass of Aeromonas hydrophila: Central composite design for optimization of process variables. J. Hazard. Mater..

[21] Akpomie KG, Dawodu FA, Adebowale KO (2015). Mechanism on the sorption of heavy metals from binary-solution by a low cost montmorillonite and its desorption potential. Alex. Eng. J..

[22] Cui L (2015). EDTA functionalized magnetic graphene oxide for removal of Pb (II), Hg (II) and Cu (II) in water treatment: adsorption mechanism and separation property. J. Chem. Eng...

[23] Mejias-Carpio IE (2014). Graphene oxide functionalized with ethylenediamine triacetic acid for heavy metal adsorption and anti-microbial applications. J. Carbon.

[24] Hosseinzadeh H, Ramin S (2018). Effective removal of copper from aqueous solutions by magnetic chitosan/graphene oxide nanocomposites modified. J Biol. Macromol..

[25] Yayayürük O, Yayayürük AE (2016). Removal of Cu (II) from water samples using Glycidyl methacrylate-based polymer functionalized with diethylenetriamine tetraacetic acid: investigation of adsorption characteristics. J. Water Air Soil Pollut..

[26] Fu W, Huang Z (2018). Magnetic dithiocarbamate functionalized reduced graphene oxide for the removal of Cu (II), Cd (II), Pb (II), and Hg (II) Ions from aqueous solution: synthesis, adsorption, and regeneration. Chemosphere.

[27] Anbia M, Amirmahmoodi S (2016). Removal of Hg (II) and Mn (II) from aqueous solution using nanoporous carbon impregnated with surfactants. Arab. J. Chem..

[28] Donohue MD, Aranovich GL (1998). Classification of Gibbs adsorption isotherms. Adv. Colloid Interface Sci..

[29] Mahmoudabadi MJ, Danesh N (2018). Gravitational search algorithm-based fuzzy control for a nonlinear ball and beam system. J. Control Decis..

[30] Ghosh A, Das P, Sinha K (2015). Modeling of biosorption of Cu (II) by alkali-modified spent tea leaves using response surface methodology (RSM) and artificial neural network (ANN). J. Appl. Water Sci..

[31] Chang Y (2016). Optimization of polyacrylonitrile–cysteine resin synthesis and its selective removal of Cu (II) in aqueous solutions. J. Chin. Chem. Lett..

